# Encapsulating Cobalt Nanoparticles in Interconnected N‐Doped Hollow Carbon Nanofibers with Enriched Co—N—C Moiety for Enhanced Oxygen Electrocatalysis in Zn‐Air Batteries

**DOI:** 10.1002/advs.202101438

**Published:** 2021-08-16

**Authors:** Qi Lu, Han Wu, Xuerong Zheng, Yanan Chen, Andrey L. Rogach, Xiaopeng Han, Yida Deng, Wenbin Hu

**Affiliations:** ^1^ School of Materials Science and Engineering Tianjin Key Laboratory of Composite and Functional Materials and Key Laboratory of Advanced Ceramics and Machining Technology of Ministry of Education Tianjin University Tianjin 300072 P. R. China; ^2^ Department of Materials Science and Engineering and Center for Functional Photonics (CFP) City University of Hong Kong 83 Tat Chee Avenue Kowloon Hong Kong SAR 999077 P. R. China; ^3^ Joint School of National University of Singapore and Tianjin University International Campus of Tianjin University Binhai New City Fuzhou 350207 P. R. China

**Keywords:** carbon nanofibers, Co—N—C moiety, high‐temperature shock, oxygen electrocatalysis, zinc‐air batteries

## Abstract

Rational design of bifunctional efficient electrocatalysts for both oxygen reduction (ORR) and oxygen evolution reactions (OER) is desirable—while highly challenging—for development of rechargeable metal–air batteries. Herein, an efficient bifunctional electrocatalyst is designed and fabricated by encapsulating Co nanoparticles in interconnected N‐doped hollow porous carbon nanofibers (designated as Co@N‐C/PCNF) using an ultrafast high‐temperature shock technology. Benefiting from the synergistic effect and intrinsic activity of the Co—N—C moiety, as well as porous structure of carbon nanofibers, the Co@N‐C/PCNF composite shows high bifunctional electrocatalytic activities for both OER (289 mV at 10 mA cm^−2^) and ORR (half‐wave potential of 0.85 V). The Co—N—C moiety in the composite can modulate the local environmental and electrical structure of the catalysts, thus optimizing the adsorption/desorption kinetics and decreasing the reaction barriers for promoting the reversible oxygen electrocatalysis. Co@N‐C/PCNF‐based aqueous Zn–air batteries (AZAB) provide high power density of 292 mW cm^−2^, and the assembled flexible ZAB can power wearable devices.

## Graphical abstract

Benefiting from the Co—N—C moiety exposure in the well‐coupled interfaces and its highly porous structure, composites based on core–shell Co nanoparticles encapsulated by N‐doped carbon distributed within porous carbon nanofibers show efficient bifunctional electrocatalytic activities. Aqueous Zn–air batteries based on those composites provide high power density and long‐term cycling, and the flexible Zn–air batteries can power digital devices.

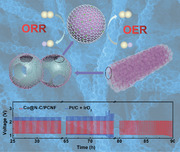

## 1 Introduction

Due to the increasing concerns on global warming and environmental pollution, research community has been motivated to explore sustainable energy technologies. As one of the promising energy storage systems, ZAB have drawn particular attention because of advantages of economical cost, safety, and high theoretical energy density.^[^ ^1^, ^2^, ^3^, ^4^, ^5^, ^6^ ^]^ The efficiency of ORR/OER plays a critical role in enhancing the performance of ZAB.^[^ ^7^, ^8^, ^9^ ^]^ While commonly employed noble metal catalysts, such as Pt, Ir, Ru, and their alloys, display satisfying OER/ORR performance, their scarce abundance, high‐cost, and lack of bifunctional features severely hampered the large‐scale application in ZAB.^[^ ^10^, ^11^, ^12^, ^13^, ^14^, ^15^, ^16^ ^]^ Therefore, efforts have been devoted to explore low‐cost, stable, and efficient bifunctional alternatives, such as transition metals (TMs) and their oxides, nitrides, and chalcogenides.

Recent studies have shown that hybrid electrocatalysts, compositing TMs or their chalcogenides with heteroatom‐doped (e.g., nitrogen‐, sulfur‐, and phosphorus‐doped) carbonaceous materials, are promising bifunctional electrocatalysts.^[^ ^17^, ^18^, ^19^ ^]^ Among them, benefiting from the high affinity of nitrogen (especially pyridinic N) toward TM atoms, composites containing N‐doped carbon and TMs (nanoparticles, nanoclusters, and even single atoms) are of particularly interest. The local electronic structure of TM, N, and C atoms can be modulated especially at the interface area, and thus the TM‐N‐C moieties were proposed to accelerate the adsorption and desorption kinetics of intermediates during OER/ORR process.^[^ ^20^, ^21^ ^]^ However, although the TM‐N‐C moieties were evidenced to be active sites for boosting the electrocatalytic properties, little has been done to analyze their local environmental and electrical structure modulation for deciphering the fundamental synergistic electrocatalysis mechanism in relation to ORR/OER. Moreover, it is still difficult to optimize the bifunctionality of the composites due to the following factors: i) low surface area of carbonaceous substrates and the limited heterointerface area between TMs nanoparticles and substrates prevent the active sites exposure; ii) low degree of graphitization of carbon could not provide high electronic conductivity; iii) poor contact between carbon and TMs limits the formation of TM‐N‐C moiety; iv) aggregation of TMs and carbon substrates at high temperature is a detrimental factor. In addition, traditional fabrication methods often involves poisonous reagents, tedious procedures, and expensive template agents, which may hinder their large‐scale application. Thus, it is crucial and imperative to develop effective strategies for fabrication of efficient and stable TM‐N‐C‐based bifunctional catalysts.^[^ ^22^, ^23^, ^24^, ^25^ ^]^

Toward this goal, HT shock technology was used in this study to encapsulate Co nanoparticles into the interconnected N‐doped hollow porous carbon nanofibers with stable core–shell structure (denoted as Co@N‐C). In this work, the Co@N‐C core–shell composites were synthesized by HT method, which show distinct advantages for controlling the crystal size, interface structure, and even the electronic structure of the composites.^[^ ^26^, ^27^ ^]^ This method is highly efficient and applicable for synthesizing various products due to the ultrafast heating/cooling rate. In the Co@N‐C/PCNF composite, the N‐doped carbon shell not only protects Co nanoparticles from alkaline electrolyte corrosion, but also provides Co—N—C moiety with Co atoms in the well‐coupled interface.^[^ ^28^, ^29^, ^30^ ^]^ The fabricated composite offers unique advantages including high graphitization degree, homogeneous distribution of Co nanoparticles without aggregation, and large specific surface area with an ultrathin carbon layer, which is beneficial for enlarging active sites exposure and promotes the mass transfer rate.^[^ ^31^, ^32^ ^]^ The experimental and theoretical results suggest that the local environmental and electrical structure of Co in the composite can be modulated in the Co—N—C moiety, which optimizes its adsorption/desorption energies and in turn boosts intrinsic OER/ORR activities. Benefiting from the synergistic effect between the Co core and N‐doped carbon shell, the Co@N‐C/PCNF composite displays outstanding reversible oxygen electrocatalytic activities with an overpotential of 289 mV at 10 mA cm^−2^ for OER and half‐wave potential of 0.85 V for ORR. Moreover, Co@N‐C/PCNF‐based AZAB show a significantly enhanced specific capacity of 292 mW cm^−2^, and flexible ZAB show excellent flexibility with long cycle life performance. Our study provides an ultrafast and facile strategy for fabricating Co—N—C moieties with well‐coupled core–shell interfaces, which offer promising applications in developing renewable energy storage systems.

## 2 Results and Discussion

**Figure 1:**
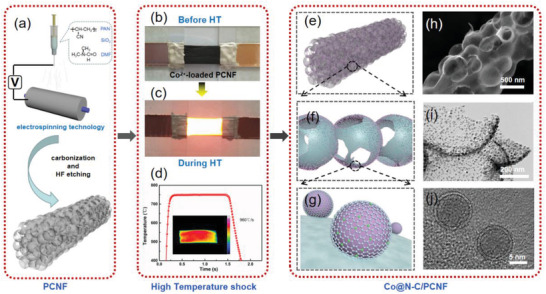
a) Schematic illustration of the HT shock synthesis of PCNF. b,c) Photographs showing the heating processes used for fabrication of Co@N‐C/PCNF. d) Temperature evolution curve during HT shock process, inset shows the temperature distribution. e–g) Structural models of different parts of Co@N‐C/PCNF, shown together with h) SEM, i) TEM, and j) HRTEM images demonstrating those selected parts of the Co@N‐C/PCNF composite.

The strategy for fabrication of Co@N‐C/PCNF composite is illustrated in **Figure** **1**. SiO_2_ inserted carbon nanofibers (SCNF) were synthesized using electrospinning technique, and PCNF were fabricated through carbonization and HF etching (Figure 1a, Figures S1 and S2, Supporting Information). Then, the PCNF were immersed into Co^2+^ solution for 30 min for ionic absorption (Figure 1b). The Co^2+^‐loaded PCNF were fixed on a glass holder, and the voltage (35 V) was applied to achieve high temperature in Ar atmosphere (Figure 1c). The temperature evolution curve during the synthesis of Co@N‐C/PCNF indicates that the heating temperature can reach 750 ℃ in 0.1 s and the cooling rate is about 960 ℃ s^−1^ (Figure 1d). The HT shock synthesis of Co@N‐C/PCNF could be finished in 2–3 s, much faster than the traditional fabrication methods. Such an ultrafast heating process leads to the reduction of Co^2+^, which self‐assemble into Co nanoparticles without further growth. We propose that the growth process of Co nanoparticles should undergo the following steps: Co‐salt decomposition at high temperature, nucleation of Co atoms, agglomeration of Co nucleus and grown into nanoparticles on the carbon substrate.^[^ ^33^ ^]^ Benefiting from the rapid cooling process, the encapsulation effect of an ultrathin carbon layer (1–3 monolayers) of the core–shell structure resulted in the isolation of nanoparticles, and thus no agglomeration occurred even at high temperature (Figure 1e‐j). The HT shock process can also improve the graphitization degree of the PCNF (Figure S3, Supporting Information), thus increasing their electronic conductivity.^[^ ^34^ ^]^ Moreover, the in situ formed highly graphitic N‐doped carbon layer in the core–shell structure is derived from the porous N‐doped PCNF and is bonded seamlessly (Figure 1g,j), thus preventing the interface resistance between the loading catalysts and the substrates, which is beneficial for accelerating the electrical conductivity during catalysis. In addition, the interconnected porous architecture of the Co@N‐C/PCNF composite provides short charge transport path and large active area, facilitating gas and mass diffusion (Figure 1f,i).

**Figure 2:**
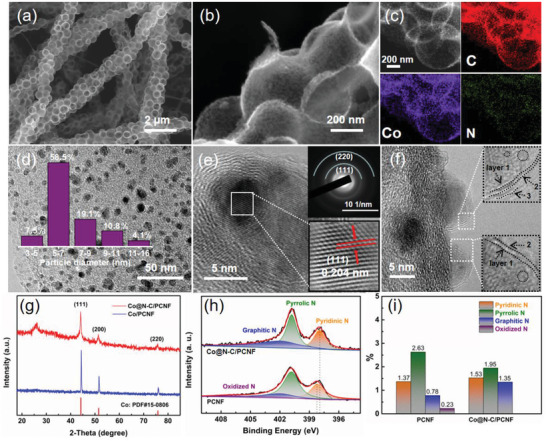
a) SEM and b) amplified SEM images of the Co@N‐C/PCNF composite. c) HAADF image and elemental mapping distribution of the Co@N‐C/PCNF composite. d) TEM image of Co@N‐C nanoparticles (insert shows the particle size distribution).e,f) HRTEM image of Co@N‐C nanoparticle; the top right and bottom right insets of (e) show SAED pattern of Co@N‐C/PCNF and Co core, respectively. g) XRD patterns, h) high‐resolution N 1s XPS spectra, and i) histograms providing contents of different chemical states of nitrogen element in PCNF and Co@N‐C/PCNF.

The morphology of the precursors of SCNF, PCNF, and the obtained Co@N‐C/PCNF composite has been characterized using scanning electron microscope (SEM) and transition electron microscope (TEM). After HF etching of SCNF, SiO_2_ spheres were decomposed and the ultrathin porous carbon shell structure was formed (Figure S2, Supporting Information), which is favorable for the mass transfer and bubble release during the catalysis. Benefiting from the porous structure, the specific surface area of PCNF is much higher than that of the non‐porous CNF (Figure S4, supporting Information). The porous structure of Co@N‐C/PCNF (**Figure** **2**a,b) was well preserved after HT shock process. High‐angle annular dark field (HAADF) image and the elemental mapping clearly reveal the homogeneous distribution of Co, N, and C elements in PCNF, and also demonstrate the even distribution of Co nanoparticles in the composite (Figure 2c and Figure S5, Supporting Information). The average diameter of the Co@N‐C nanoparticles is around 5–7 nm (Figure 2d). The high‐resolution TEM (HRTEM) images (Figure 2e,f) unveiled the microstructure of core–shell Co@N‐C/PCNF composites. The N‐doped carbon shells were coated on the surface of Co nanoparticles, preventing the agglomeration and corrosion of Co core during electrocatalysis.^[^ ^35^ ^]^ The lattice fringe of the N‐doped carbon shell can be clearly observed, suggesting its high graphitization degree and good electrical conductivity. It should be noted that the thin carbon layer typically forms on the Co particle surface, which will allow the efficient gas/mass transfer and also benefit the exposure of the catalytically active sites. The lattice spacing of the core part is 0.204 nm, corresponding to the (111) plane of Co (Figure 2e). Abundant lattice distortions of Co nanoparticles can be observed in black dotted areas in Figure 2f, which could potentially increase the intrinsic activity and thus facilitate the chemisorption and desorption of oxygen‐containing intermediates, enhancing the apparent catalytic activity. The selected area electron diffraction (SAED) pattern confirmed the phase structure of Co nanoparticles with two diffraction rings corresponding to the crystal planes of (220) and (111) in Co (Figure 2f). For comparison, we also prepared Co/PCNF composite without core–shell structure (Figure S6, supporting Information) using heat treatment method, in which Co nanoparticles severely agglomerated with size of ≈100 nm.

As shown in the X‐ray diffraction (XRD) pattern (Figure 2g), diffraction peaks at 44.23˚, 51.53˚, and 75.86˚ can be assigned to the Co nanoparticles in both Co@N‐C/PCNF and Co/PCNF, corresponding to the (111), (200), and (220) planes (JCPDS No. 89–4307), which is consistent with the HRTEM and SAED data. The broadened half‐width of diffraction peaks for Co@N‐C/PCNF confirm that Co nanoparticles are smaller in that case, compared to those in Co/PCNF. The additional XRD peak at 26.23˚ is attributed to the (002) plane of graphitized carbon, suggesting a higher graphitization degree of carbon in Co@N‐C/PCNF after HT shock, which is consistent with the Raman data (Figure S3, Supporting Information). Moreover, we systematically investigated the influence of the Co mass loading on the morphology variation and electrochemical performance of resulting composites (Figures S7 and S8, Supporting Information). With the increase of the Co^2+^ precursor concentration from 50 to 200 mm, the mass loading of Co nanoparticles increased and the OER/ORR performance was optimized. However, when further increasing the concentration to 300 mm, the surface of the composite became uneven and the OER/ORR performance became worse.^[^ ^36^, ^37^, ^38^, ^39^ ^]^ This can be caused by the agglomeration of nanoparticles and thus decreasing the active sites exposure. Therefore, we selected Co^2+^ precursors concentration of 200 mm to fabricate all composites for further studies. In addition, we also synthesized Ni@N‐C/PCNF and CoNi@N‐C/PCNF electrocatalysts (Figure S9, Supporting Information) using the HT shock method with similar experimental parameters. These results demonstrate that this method is a general strategy for preparing carbon‐coated transition metal nanoparticles as composite electrocatalysts.

Based on the above results, we hypothesize that the HT shock and the interface effects in the core–shell structure of Co@N‐C/PCNF would result in the local electrical modulation and chemical structure variation, which could potentially influence the electrocatalytic performance of the resulting composites. X‐ray photoelectron spectroscopy (XPS) was performed to provide insights into the chemical states. The XPS is a powerful technique to evaluate the chemical states of elements on the surface of catalysts. The peaks of high‐resolution N 1s spectra in both Co@N‐C/PCNF and PCNF were deconvoluted into different nitrogen configurations (pyridinic N at 398.1 eV, pyrrolic N at 400.7 eV, graphitic N at 401.8 eV, and oxidized N at 403.5 eV), and their contents were quantitatively estimated. Comparing to the N 1s spectrum in PCNF, the pyridinic N and pyrrolic N in Co@N‐C/PCNF display an obvious negative shift (≈0.4 eV). Moreover, the appearance of high‐valence Co (Figure S10a, Supporting Information) demonstrates that the Co species at the core–shell interfaces were partly oxidized due to the electron transfer from Co to N in the N‐doped carbon support.^[^ ^40^ ^]^ The presence of C‐N bond in both Co@N‐C/PCNF and PCNF points out on successful doping of N into carbon (Figures S10b and S11, Supporting Information).^[^ ^41^ ^]^ According to the deconvolution results (Figure 2i), as compared to PCNF, the content of graphitic N and pyridinic N in Co@N‐C/PCNF increased from 0.78% to 1.37% and from 1.35% to 1.53%, respectively; however, pyrrolic N decreased from 2.63% to 1.95%, demonstrating that both pyrrolic N and oxidized N were reduced and the graphitization degree was improved as a result of HT shock process, which is consistent with the XRD results (Figure 2g). Therefore, the presence of Co—N—C moiety and the electron transfer in the composite have been confirmed by the XPS results. However, local structural variation of the Co—N—C moiety in the core–shell structure needs to be precisely explored further, which may offer fundamental insights for designing and developing highly efficient TM‐N‐C‐based catalysts.

**Figure 3:**
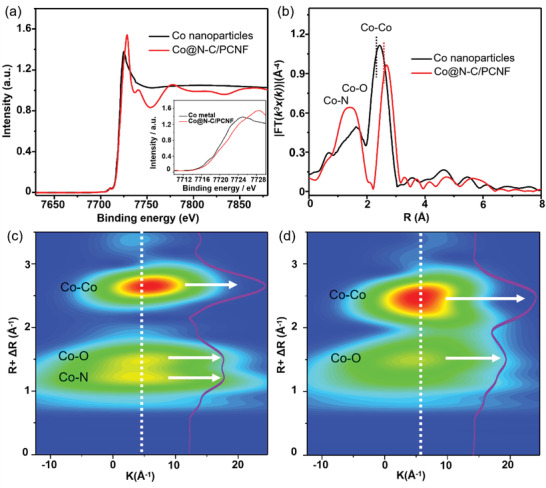
a) EXAFS spectra of the Co K‐edge in Co nanoparticles and Co@N‐C/PCNF, inset shows amplified spectra of the K‐edge. b) R‐space spectra of the Co K‐edge in Co nanoparticles and Co@N‐C/PCNF. MWT contour plots of the Co K‐edge EXAFS of c) Co@N‐C/PCNF and d) Co nanoparticles. White arrows show signals from Co‐Co, Co‐O and Co‐N bonds; purple lines are profile curves corresponding to the white lines in Z‐direction.

To further trace the local environmental and the electrical structure variation of Co—N—C moiety in the core–shell structure, we collected extended X‐ray absorption fine structure (EXAFS) spectra. Co nanoparticles in the PCNF (without N‐C coating on the surface, see Figure S6, Supporting Information) were used for comparison. EXAFS spectra (**Figure** **3**a) show that, as compared to Co nanoparticles, the K‐edge of Co in Co@N‐C/PCNF shifted toward higher binding energy, demonstrating the oxidation of Co in Co@N‐C/PCNF composites, which is consistent with the XPS results. Furthermore, we performed the Fourier transform (FT) based on the k^3^‐weighted *χ*(k)‐function curves of the Co K‐edge EXAFS spectra. R‐space spectra (Figure 3b) show that, for Co nanoparticles, the peak located at 2.37 Å can be assigned to Co–Co bonds. The Co–Co peak in Co@N‐C/PCNF shifted toward higher binding energy direction comparing to that of Co nanoparticles. For Co@N‐C/PCNF, due to the overlap between Co‐O and Co‐N peaks, it is difficult to classify the two peaks in the R‐space spectra. This should be caused by two factors: 1) the two peaks of Co‐O and Co‐N stand too close to be classified and form a broad overlapped peak at ≈1.52 Å; 2) the multiple scattering phenomenon from different layers in the lattice structure. However, the broad peak demonstrates the formation of Co–N species in Co@N‐C/PCNF.^[^ ^42^ ^]^

To further unveil the local bonding environment variation of Co in the composites, Morlet wavelet transform (MWT) was performed. MWT is a powerful method to identify the local environment of metal complexes by directly visualizing all the EXAFS signal contributions from neighboring atoms. It can also efficiently identify the overlapping signals in the EXAFS spectra, thus avoiding potential influence from the multiple scattering of different layers in the lattice structure.^[^ ^43^, ^44^, ^45^ ^]^ Obviously, two maxima corresponding to Co–O and Co–N coordination appeared in the MWT contour plot of Co@N‐C/PCNF (Figure 3c), confirming the oxidation of Co in the Co—N—C moiety. Purple lines are the profile curves of the white lines in Z‐direction (Figure 3c,d). Obviously, Co‐N bonds appeared in the Co@N‐C/PCNF composite, demonstrating the well‐coupling interface formation in the composite. Thus, EXAFS spectra and the corresponding R‐space spectra and MWT data demonstrate that the local environmental coordination in the Co@N‐C/PCNF composites is indeed modulated by the core–shell interface structure, which may potentially enhance the electrocatalytic activity of these catalysts.

**Figure 4:**
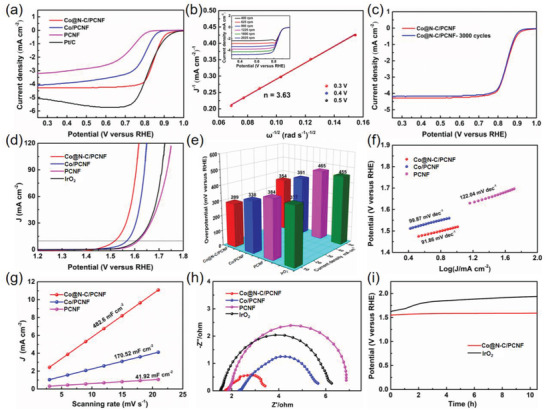
a) ORR LSV curves of Co@N‐C/PCNF, Co/PCNF, PCNF, and Pt/C tested at 5 mV s^−1^ with a rotation speed of 1600 rpm. b) Fitted K–L plots of Co@N‐C/PCNF based on the LSV curves at 0.3, 0.4, and 0.5 V, inset shows ORR polarization curves of Co@N‐C/PCNF at different rotating speeds. c) ORR polarization curves of Co@N‐C/PCNF before and after 3000 CV cycles. d) OER LSV curves of four listed catalysts at 5 mV s^−1^. e) OER overpotentials of four listed catalysts at current densities of 10 mA cm^−2^ and 50 mA cm^−2^. f) Tafel slopes, g) *C*_dl_ values, and h) EIS Nyquist plots of Co@N‐C/PCNF, Co/PCNF, and PCNF. i) Chronopotentiometry response of Co@N‐C/PCNF and IrO_2_ at current density of 10 mA cm^−2^.

We further explored reversible oxygen electrocatalytic properties of the Co@N‐C/PCNF composite. As shown in the ORR linear sweep voltammograms (LSV) curves (**Figure** **4**a and Table S1, Supporting Information), the half‐wave potential and limiting diffusion current density of Co@N‐C/PCNF are 0.85 V and 4.32 mA cm^−2^, respectively, approaching the corresponding values of the 20 wt% Pt/C catalyst (0.83 V vs RHE and 5.01 mA cm^−2^, respectively). Moreover, the Co@N‐C/PCNF shows a higher ORR activity than Co/PCNF (0.78 V and 4.1 mA cm^−2^), PCNF (0.71 V and 3.3 mA cm^−2^), and other non‐precious‐metal‐based catalysts (Table S2, Supporting Information). Fitted Koutecky–Levich (K–L) plots (Figure 4b) show that the electron transfer number (*n*) for Co@N‐C/PCNF is 3.63, suggesting that these composites possess the first‐order reaction kinetics with quasi‐4e pathway for ORR catalysis.^[^ ^19^ ^]^ Moreover, the ORR LSV curves of Co@N‐C/PCNF show negligible decay even after 3000 continuous cycles (Figure 4c), demonstrating superior ORR durability of this composite which is important for large‐scale applications.

In the context of developing efficient bifunctional catalysts, OER LSV curves of the produced catalysts were tested in N_2_‐saturated 1.0 m KOH electrolyte. The Co@N‐C/PCNF exhibits OER overpotentials of 289 and 354 mV at current densities of 10 and 50 mA cm^−2^, respectively, which is superior to that of Co/PCNF (338/391 mV), PCNF (384/465 mV) and commercial IrO_2_ (377/455 mV) (Figure 4d,e and Table S1, Supporting Information) and comparable to the recently reported TM‐based catalysts (Table S3, Supporting Information). As shown in Figure 4f, Co@N‐C/PCNF also has a smaller Tafel slope (91.8 mV dec^−1^) than that of Co/PCNF (99.8 mV dec^−1^) and PCNF (149.2 mV dec^−1^), indicating its better OER reaction kinetics. Moreover, electrochemically active surface area (ECSA) of the catalysts was evaluated by the double layer capacitance (*C*_dl_), which was tested by measuring CV curves at different scanning rates (Figure S12, Supporting Information). Co@N‐C/PCNF has higher *C*_dl_ (482.6 mF cm^−2^) than that of Co/PCNF (170.52 mF cm^−2^) and PCNF (41.92 mF cm^−2^), suggesting its larger active sites exposure (Figure 4g). In addition, the electrochemical impedance spectroscopy (EIS) of the catalysts has been performed (Figure 4h), and the resulting Nyquist plots showed that Co@N‐C/PCNF possess the smallest charge transfer resistance (*R*_ct_, 1.69 Ω) compared to the counterparts of Co/PCNF (3.36 Ω) and PCNF (5.01 Ω), which demonstrates faster catalytic kinetics for Co@N‐C/PCNF. We also studied the catalytic durability of Co@N‐C/PCNF at 10 mA cm^−2^, which shows no obvious degradation in the chronopotentiometry response for 10 h, which is even more stable than that of IrO_2_ (Figure 4i). TEM, HRTEM, and XPS were performed on the catalysts after long‐term OER reaction. As shown in Figure S13a–c, Supporting Information, the morphology and microstructure of the core–shell composites are kept very well, demonstrating the outstanding stability of the catalysts. The XPS spectra (Figure S13d) shows that only a small peak of Co 2p can be assigned to Co^2+^, which should be caused by the long‐term exposure to air. The fitting peaks in N 1s spectra belongs to pyridinic N, pyrrolic N and graphitic N (Figure S13e, Supporting Information), respectively, which is the same as that before OER process. These results confirmed the outstanding stability of the Co@N‐C/PCNF. The aforementioned superior bifunctional catalytic performance of Co@N‐C/PCNF should be attributed to the synergistic effect of the Co—N—C moiety, which accelerates the catalytic kinetics of both OER and ORR.

**Figure 5:**
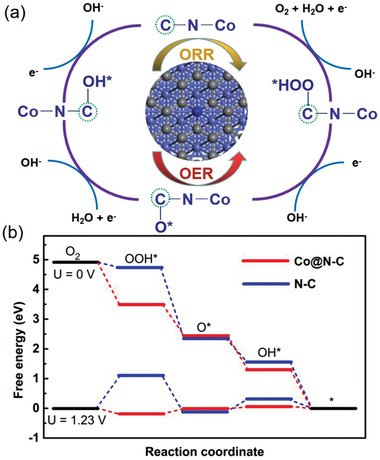
a) The ORR (shown clockwise) mechanisms over Co@N‐C. b) The calculated free energy diagrams for the ORR pathway of Co@N‐C and N‐C catalysts.

To gain deeper insights on the crucial role of Co—N—C moiety structure in Co@N‐C/PCNF on synergistic electrocatalysis for OER and ORR, density functional theory (DFT) calculations were performed to reveal the energetically favorable pathway for ORR catalysis on Co—N—C moiety, and compared with the N‐doped carbon shell (N‐C) as a reference from previous literature (**Figure** **5** and Figure S14, Supporting Information).^[^ ^18^ ^]^ The proposed ORR/OER mechanisms of Co@N‐C/PCNF catalyst are illustrated in Figure 5a. The catalytic active sites were selected by pre‐adsorbing oxygen intermediates (O*, OH*, and OOH*) on Co, N, and C sites. The reaction intermediates are more inclined to be adsorbed on the C site of Co—N—C moiety, which is consistent with other related reports.^[^ ^46^ ^]^ When no potential is applied (U = 0 V), the catalysts exhibit a downhill pathway for ORR process (Figure 5b). Therefore, the rate‐determining step (RDS) should be the minimum free energy change (Δ*G*) step. The RDS for Co@N‐C and N‐C are OOH* to O* and O_2_ to OOH*, and the corresponding Δ*G* values are 1.077 and 0.153 eV, respectively. Therefore, the Co@N‐C catalyst displays larger Δ*G* value for RDS at 0 V, and lower Δ*G* values for |OOH*|, |O*|, and |OH*| when the equilibrium potential (1.23 V) is applied. Thus, both experimental and theoretical results point on the critical role of the synergistic effect of the Co—N—C moiety in the core–shell structure of Co@N‐C/PCNF, which could potentially reduce the ORR barrier and accelerate the catalytic kinetics.

Based on the above chemical and electrochemical characteristics, and DFT computational insights, superior ORR/OER properties of the Co@N‐C/PCNF composite can be attributed to the following factors: i) synergistic effect and highly intrinsic activity of Co—N—C moiety in the core–shell structure; ii) enlarged active sites exposure and enhanced electric conductivity ensured by the well‐coupled interfaces and the porous substrates. Moreover, the encapsulated core–shell structure could protect Co nanoparticles from oxidation and corrosion, thus enhancing the durability of the composite in alkaline electrolytes.

**Figure 6:**
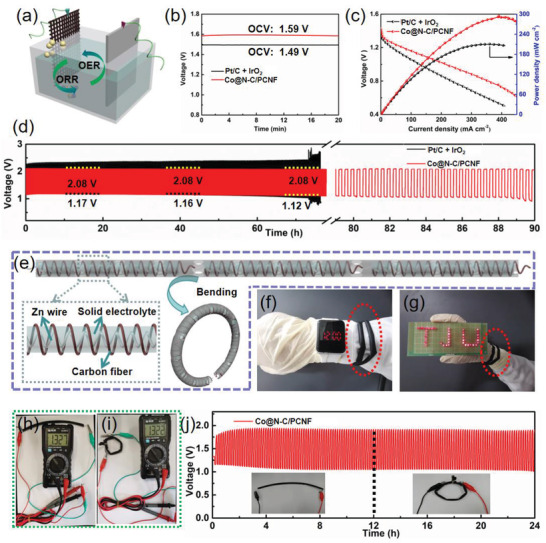
a) Schematic configuration of AZAB. b) Open‐circuit plots of Co@N‐C/PCNF and noble‐metal‐based AZAB. c) Corresponding discharge polarizations and power density curves. d) Long‐term cycling performance of the two AZABs at a current density of 5 mA cm^−2^. e)Schematic configuration of flexible ZAB. Photographs of f) digital watch and g) an LED screen that were powered by three Co@N‐C/PCNF‐based flexible ZABs interconnected in series. Photographs showing open‐circuit potentials of flexible ZAB in h) straight state and i) after 360˚ bending. j) Long‐term cycling performance of Co@N‐C/PCNF‐based flexible ZAB at a current density of 5 mA cm^−2^ for the two different bending angles (straight and 360°).

Outstanding bifunctional ORR/OER properties of Co@N‐C/PCNF composite made them useful components of rechargeable AZAB. Mixed noble metal catalysts of Pt/C+IrO_2_ were used as cathodes for comparison. AZAB were assembled using Zn foil as anode and the mixture of 6.0 m KOH and 0.2 M Zn(CH_3_COO)_2_ as electrolyte (**Figure** **6**a).^[^ ^47^, ^48^ ^]^ The Co@N‐C/PCNF‐based AZAB exhibited an open‐circuit voltage (OCV) of 1.59 V (Figure 6b), a power density of 292 mW cm^−2^ (Figure 6c), and the specific capacity of 322.3 mAh g^−1^_Zn_ (Figure S15, Supporting Information) which is higher than that of Pt/C+IrO_2_‐based AZAB (1.49 V, 210.6 mW cm^−2^, and 274.0 mAh g^−1^_Zn_).^[^ ^49^ ^]^ Compared to that of noble metals, the higher power density of AZAB based on Co@N‐C/PCNF suggests the efficient ORR catalytic capability, highlighting its great potential for practical device applications. Moreover, the Co@N‐C/PCNF‐based AZAB showed better cycling stability (up to 285 cycles) than that of Pt/C + IrO_2_ (Figure 6d), which is consistent with the OER and ORR results. This confirmed the excellent catalytic activity and stability of the Co@N‐C/PCNF composite as air cathodes in the rechargeable AZAB.

In the context of developing flexible solid‐state power sources for the application in wearable devices, flexible ZAB were also assembled using Co@N‐C/PCNF composite as cathodes, Zn wire as anodes, and the modified polyvinyl alcohol (PVA) as a solid‐state electrolyte (Figure 6e).^[^ ^50^ ^]^ Three series‐connected flexible ZAB could be bend into two circles on the wrist and are able to power the digital watch (Figure 6f) and the light‐emitting diode (LED) screen (Figure 6g). Moreover, flexible ZAB exhibited an OCV of above 1.32 V and remained stable even at 360˚ bending state (Figure 6h,i and Figure S16, Supporting Information). Remarkably, in both straight and bending states (for more than 24 h and about 150 cycles), no obvious performance loss was observed for Co@N‐C/PCNF in the charge/discharge cycling process (Figure 6j). Compared with other non‐precious‐metal‐based catalysts, Co@N‐C/PCNF‐based flexible ZAB displays comparable or even better cycling stability (Table S4, Supporting Information), indicating its potential application in the portable and wearable devices.

## 3 Conclusions

We have used ultrafast (2–3 s) HT shock treatment for fabrication of the Co@N‐C/PCNF composite. Benefiting from the formation of Co—N—C moiety and the synergistic effect between Co and N‐C sites at the interface of the core–shell structure, Co@N‐C/PCNF composite shows outstanding reversible oxygen electrocatalytic activities for both OER (overpotential of 289 mV at 10 mA cm^–2^) and ORR (half‐wave potential of 0.85 V). Experimental and theoretical data collectively demonstrate that the chemical states and electronic structure variation at the interface of Co—N—C moiety synergistically reduce the energetic barrier for adsorbing the oxygen intermediates and speed up the reaction kinetic, substantially enhancing the bifunctional electrocatalytic activities. Moreover, with the Co@N‐C/PCNF composite employed as air electrodes, aqueous, and flexible ZABs show efficient specific capacity and long‐term durability. This work provides a convenient route toward efficient metal‐carbon‐based oxygen electrocatalysts, as well as understanding of synergistic effects for boosting the oxygen catalytic performance.

## Conflict of Interest

The authors declare no conflict of interest.

## Supporting information

Supporting Information

## Data Availability

Research data are not shared.
