# Supplementary material for: Encapsulating Cobalt Nanoparticles in Interconnected N‐Doped Hollow Carbon Nanofibers with Enriched Co—N—C Moiety for Enhanced Oxygen Electrocatalysis in Zn‐Air Batteries
Source: Adv Sci (Weinh). 2021 Aug 16;8(20):2101438. doi: 10.1002/advs.202101438 (PMC8529470; doi:10.1002/advs.202101438)
Supplement: Supplementary file 1 — Supporting Information [file ADVS-8-2101438-s001.pdf]

© 2021 Wiley-VCH GmbH

ADVANCED SCIENCE
Open Access

Supporting Information

for *Adv. Sci.,* DOI: 10.1002/advs.202101438

Encapsulating Cobalt Nanoparticles in Interconnected N-doped Hollow Carbon Nanofibers with Enriched Co-N-C Moiety for Enhanced Oxygen Electrocatalysis in Zn-air Batteries

Qi Lu, Han Wu, Xuerong Zheng, Yanan Chen,* Andrey L. Rogach, Xiaopeng Han,* Yida Deng,* Wenbin Hu,*

Encapsulating cobalt nanoparticles in interconnected N-doped hollow carbon nanofibers with enriched Co-N-C moiety for enhanced oxygen electrocatalysis in Zn-air batteries

Qi Lu,[1] *Han Wu,*[1] *Xuerong Zheng,*[1,2*] *Yanan Chen,*[1*] *Andrey L. Rogach,*[2] *Xiaopeng Han,*[1*] *Yida Deng,*[1*] *Wenbin Hu,*[1, 3]

Q. Lu, H. Wu, Dr. X. Zheng, Prof. X. Han, Prof. Y. Chen, Prof. Y. Deng, Prof. W. Hu
School of Materials Science and Engineering, Tianjin Key Laboratory of Composite and Functional Materials, and Key Laboratory of Advanced Ceramics and Machining Technology of Ministry of Education, Tianjin University, Tianjin 300072, P. R. China
E-mail: xrzh@tju.edu.cn; xphan@tju.edu.cn; yananchen@tju.edu.cn; yida.deng@tju.edu.cn
Dr. X. Zheng, Prof. A. L. Rogach
Department of Materials Science and Engineering, and Center for Functional Photonics (CFP), City University of Hong Kong, 83 Tat Chee Avenue, Kowloon, Hong Kong S.A.R., P. R. China
Prof. W. Hu
Joint School of National University of Singapore and Tianjin University International Campus of Tianjin University, Binhai New City, Fuzhou 350207, P. R. China

Experimental Section.

***Synthesis of SiO_2 spheres*:** SiO_2 spheres were synthesized according to the previous report with some modifications.[1] Solution A: 4.2 mL TEOS (Tetraethyl orthosilicate) was added into 25 mL ethanol at room temperature and stirred for 1 h; solution B: 4 mL $NH_3 \cdot H_2O$ was added into a solution containing 25 mL ethanol and 14.3 mL water at room temperature and stirred for 1 h. Then solution A was quickly poured into solution B, and the mixture was stirred for 1 h. SiO_2 spheres were obtained after purification with ethanol and ultrapure waterand dried at 60 ℃ for 8 h.

***Synthesis of hollow porous carbon nanofibers (PCNF)*:** 1 g PAN (Polyacrylonitrile) was dissolved in 10 g DMF (N,N-Dimethylformamide), then 0.4 g SiO_2 spheres were added and stirred for 12 h. The precursor solution was electrospun using an electrospinning machine with the following parameters: 25 kV working voltage, 15 cm of the distance needle tip collector, 1 mL h^{-1} solution flow rate. The obtained film was heated to 270 °C and maintained for 1 h. After that, the fiber mat was heated at 1000 °C in a nitrogen atmosphere for 0.5 h to produce the SCNF film. The SCNF film was immersed into 10 wt% HF acid solution for 12 h, washed using deionized water, and dried in the air at 60 °C for 24 h to obtain PCNF membranes, which were cut into 2 cm × 1 cm pieces for further application..

Synthesis of Co@N-C nanoparticles on PCNF by high temperature (HT) shock technique, resulting in Co@N-C/PCNF composite: Cobalt (II) acetate tetrahydrate (200 mM) was dissolved in water; PCNF membrane was immersed in this solution for 30 min and then dried in the air at 60 °C for 1 h. A freestanding PCNF membrane with cobalt salt (Co^{2+}-loaded PCNF) was mounted on a glass slide substrate. Copper ribbons were employed as wires connected to the external circuit. Silver paste was applied to both ends of the film to connect the film with copper ribbons. The HT shock treatment was performed by applying 35 V voltage to the pre-mounted film in an argon-filled glove box for 2-3 sec.[2]

***Synthesis of Co/PCNF*:** Cobalt (II) acetate tetrahydrate (200 mM) was dissolved in water; PCNF membrane was immersed in this solution for 30 min and dried in the air at 60 ºC for 1 h. Then, the composite was heat treated to 900 ºC (5 ºC min^{-1}) for 2 h in Ar atmosphere.

Materials characterization: The morphology and structure of the products were characterized using scanning electron microscope (SEM; JSM-7800F) equipped with energy dispersive X-ray spectroscopy (EDX). Transmission electron microscopy (TEM) was carried out on a JEM-ARM200F instrument. Raman spectra were measured using Confocal Raman Microscopy (LabRAM HR Evolution) with the excitation laser line at 532 nm. Light microscopy images were obtained using SOPTOP-BH200m. X-ray photoelectron spectra (XPS) were collected on a ThermoFischer (ESCALAB 250XI) instrument. Brunauer-Emmett-Teller (BET) surface areas was estimated using nitrogen physisorption (ASAP2460). Temperature evolution during the synthesis of Co@N-C/PCNF was measured using ImageIR8355BB high-speed thermal imaging camera. EXAFS spectra were performed in transmission mode at the Beijing Synchrotron Radiation Facility (SSRF). Fortran-based HAMA code to do the Morlet wavelet transforms (MWT) of k^2 weighted EXAFS spectra[3,4]. For appropriate resolution in the R- and K-space, a wavelet parameter combination of k = 15 and σ = 2 was well suited to discriminate atomic contributions in the Fourier transforms of Co K-edge EXAFS spectra.

***Electrochemical Measurements*:** For preparing the slurry, 7 mg of composites and 3 mg of carbon black were dispersed in a mixture of 100 μL Nafion (5%) solution and 900 μL isopropyl alcohol and sonicated for 30 min. All electrochemical measurements were conducted on an electrochemical workstation (CH Instruments 660E) using standard three-electrode system. For catalyzing ORR, the slurry was dropped onto a polished glassy carbon electrode with a mass loading of 0.35 mg cm^{-2} and was used as the working electrode. A graphite electrode and a saturated calomel electrode were used as the counter electrode and reference electrode, respectively. O_2-saturated 0.1 M KOH solution was used as the electrolyte. For catalyzing OER, the slurry was dropped on a carbon cloth as the working

electrode with a catalyst loading of 2.1 mg cm^{-2}. 1.0 M KOH solution was used as the electrolyte. Linear sweep voltammetry (LSV) of ORR or OER was conducted at 5 mV s^{-1}. The potentials were adjusted to the reversible hydrogen electrode (RHE) based on the following equation:

$$E\ (\text{vs. RHE}) = E\ (\text{vs. SCE}) + 0.059 \times \text{pH} + 0.241\ \text{V} \quad (1)$$

Electrochemical impedance spectroscopy (EIS) was measured in a frequency range from 100 kHz to 0.01 Hz at 0.53 V.

Assembly of aqueous zinc-air batteries (AZAB)**:** AZAB were assembled using Zn as anode, as-prepared catalyst as cathode, and the mixture of 6.0 M KOH and 0.2 M $Zn(CH_3COO)_2$ as electrolyte. The mixture of commercial Pt/C and IrO_2 (mass ratio of 1:1) was used as a reference catalyst. The properties of all AZAB were evaluated under O_2-saturated conditions.[5]

Assembly of flexible zinc-air batteries (ZAB): Flexible ZAB were assembled using Zn wire as anodes, the as-prepared catalysts coated carbon fiber cloth as cathodes, and polyvinyl alcohol (PVA) gel film as solid electrolyte. The zinc wire was rolled into spring shape. The solid polymer electrolyte was prepared as follows: 3 g of polyvinyl alcohol was fully dissolved in 27 mL distilled water at 90 ^{o}C with magnetic stirring for 2 h, then 6 mL KOH solution (containing 3 g KOH) was added and stirred for 40 min to obtain a transparent mixed solution. The mixed liquid was transferred to a container with a zinc electrode and frozen. After freezing, the catalyst-carbon fiber cloth was wrapped on the spring-like Zn wire electrode wrapped by solid electrolyte, and then the two electrodes are packaged with heat shrinkable film to finally assemble the flexible ZAB. To assess the electrochemical properties of flexible ZAB, the charge/discharge curves and cycling stability measurements were tested under 1.0 mA cm^{-2}. The charge/discharge performance, open circuit and cycling ability were carried out on a LAND-CT2001A testing device at room temperature.

DFT calculations: First principles calculations in the framework of density functional theory (DFT) were carried out based on the Cambridge Sequential Total Energy Package known as CASTEP.[6] The exchange–correlation functional under the generalized gradient approximation (GGA)[7] with norm-conserving pseudopotentials and Perdew–Burke–Ernzerhof functional was adopted to describe the electron–electron interaction.[8] An energy cutoff of 750 eV was used and a k-point sampling set of 7 x 7 x 1 were tested to be converged. A force tolerance of 0.01 eV $Å^{-1}$, energy tolerance of $5.0x10^{-7}$eV per atom and maximum displacement of $5.0x10^{-4}$ Å were considered. The vacuum space along the Z direction was set to be 15 Å, which was enough to avoid interaction between the two neighboring images. N-doped graphene on Co (111) surface has been considered. -OH, -O and -OOH groups were adsorbed on the surface. The Grimme method for DFT-D correction was used for all calculations.[9] The electrochemical model of ORR developed by Nørskov[10] can be divided into four one-electron reactions:

$$O_2 + * + (H_2O + e^-) \rightarrow *OOH + OH^- \quad (S1)$$

$$*OOH + e^- \rightarrow *O + OH^- \quad (S2)$$

$$*O + (H_2O + e^-) \rightarrow *OH + OH^- \quad (S3)$$

$$*OH + e^- \rightarrow * + OH^- \quad (S4)$$

Gibbs free energy changes for steps S5-S8 can be calculated by:

$$\Delta G_1 = 4.92\text{eV} - \Delta G_{*OOH} \quad (S5)$$

$$\Delta G_2 = \Delta G_{*OOH} - \Delta G_{*O} \quad (S6)$$

$$\Delta G_3 = \Delta G_{*O} - \Delta G_{*OH} \quad (S7)$$

$$\Delta G_4 = \Delta G_{*OH} \quad (S8)$$

where the sum of ΔG_{1-4} is fixed to the negative of the experimental Gibbs free energy of formation of two water molecules ($-2\,\Delta_{H_2O}^{exp}$ = 4.92 eV). The over-potential of ORR is determined by the following equations:

$$\eta^{ORR} = 1.23 - U_{ORR} \quad (S9)$$

$$U_{ORR} = -\text{Max}\,(\Delta G_{*OOH} - 4.92\text{ eV}, \Delta G_{*O} - \Delta G_{*OOH}, \Delta G_{*OH} - \Delta G_{*O}, -\Delta G_{*OH})/e \quad (S10)$$

Free energy change ΔG of the reaction was calculated as the difference between the free energies of the initial and final states as shown below:[11-12]

$$\Delta G = \Delta E + \Delta ZPE - T\Delta S \quad (2)$$

where ΔE is the energy change between the reactant and product obtained from DFT calculations; ΔZPE is the change of zero point energy; T equal to 300 K and ΔS denote temperature and change of entropy, respectively. T = 300K was considered.

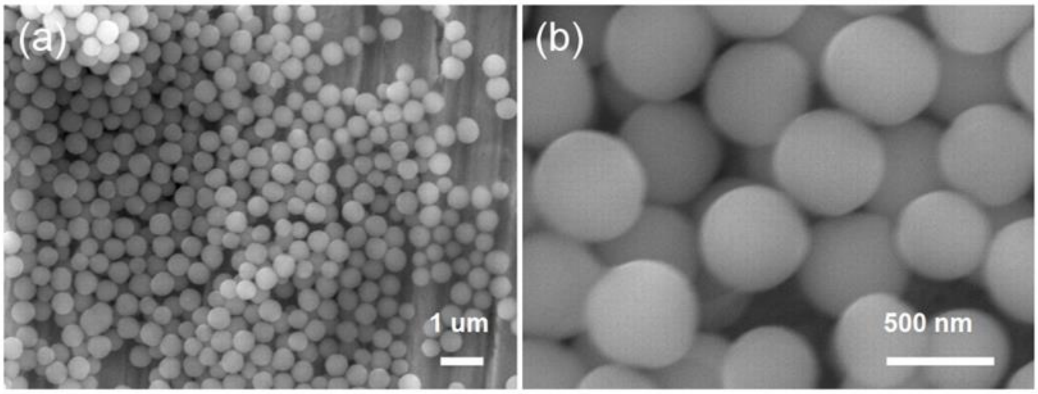

Figure S1. (a) SEM, and (b) amplified SEM images of SiO_2 spheres.

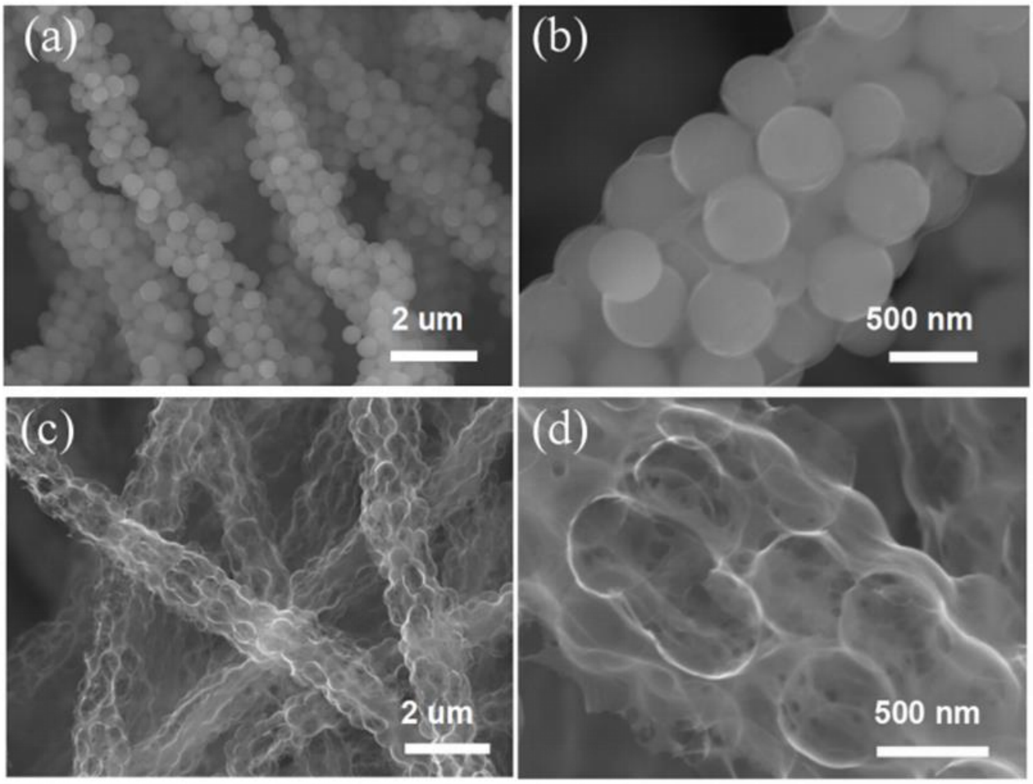

Figure S2. SEM images of (a-b) SCNF and (c-d) PCNF.

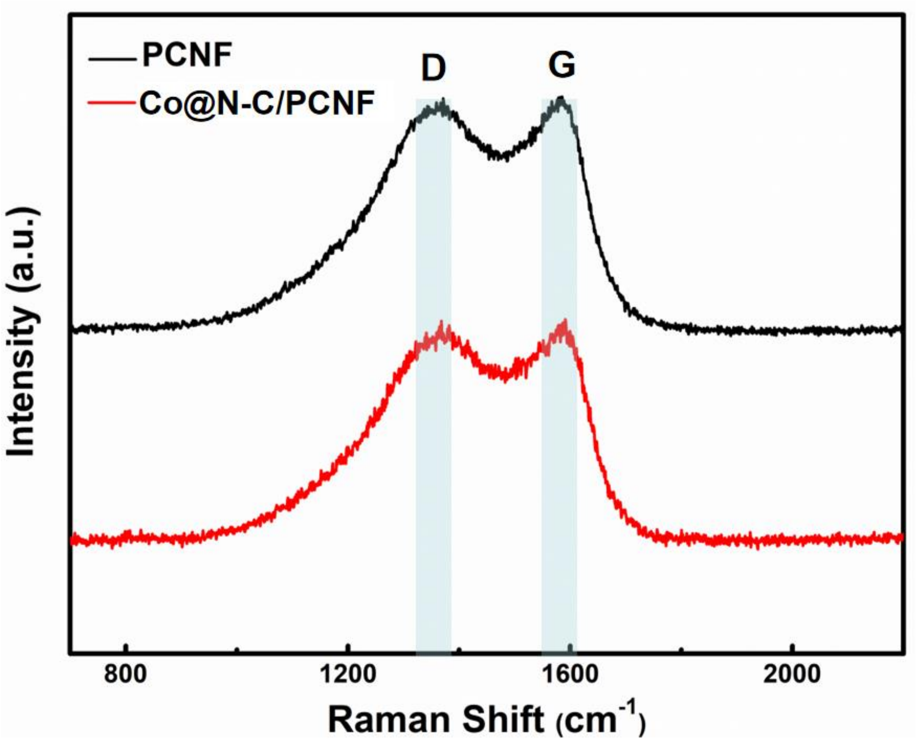

Figure S3. Raman spectra (two peaks for D and G bands) of PCNF and Co@N-C/PCNF.

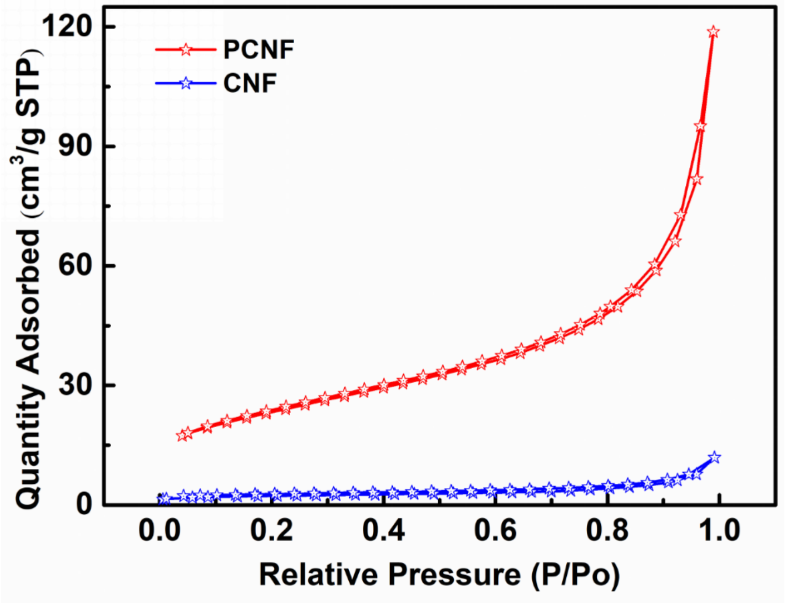

Figure S4. N_2 adsorption-desorption isotherm of CNF and PCNF.

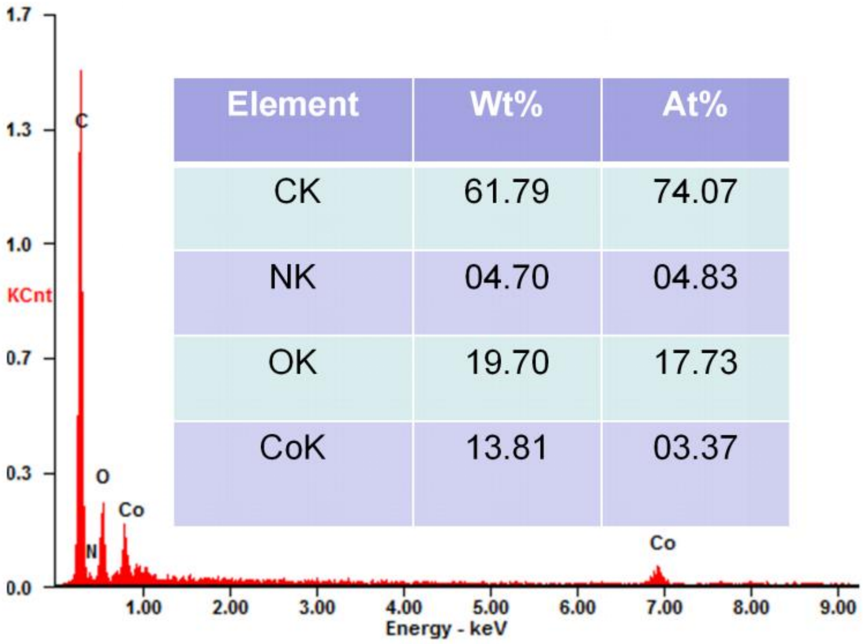

Element	Wt%	At%
CK	61.79	74.07
NK	04.70	04.83
OK	19.70	17.73
CoK	13.81	03.37

Figure S5. EDS spectrum of Co@N-C/PCNF, with a table showing constituting elements.

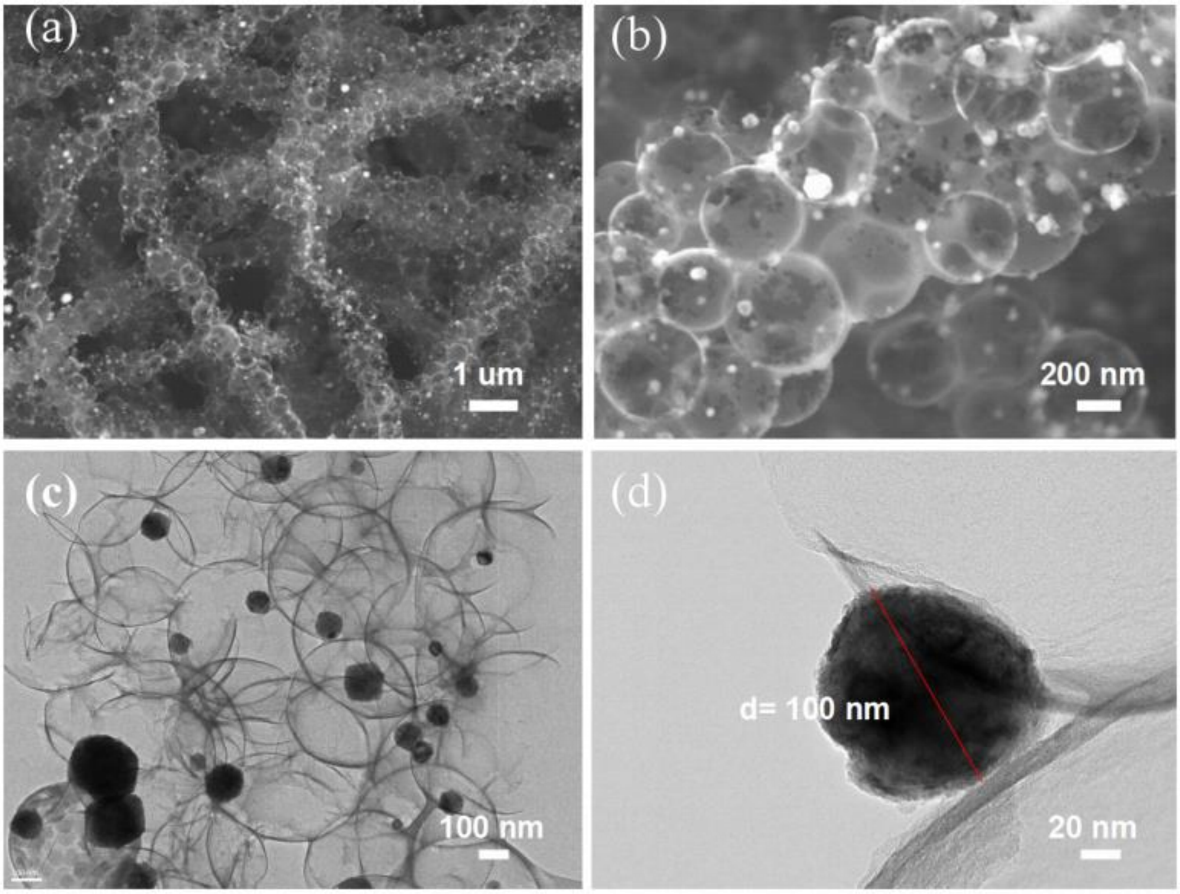

Figure S6. (a) SEM, (b) amplified SEM images of Co/PCNF. (c) TEM, and (d) amplified TEM images of Co/PCNF.

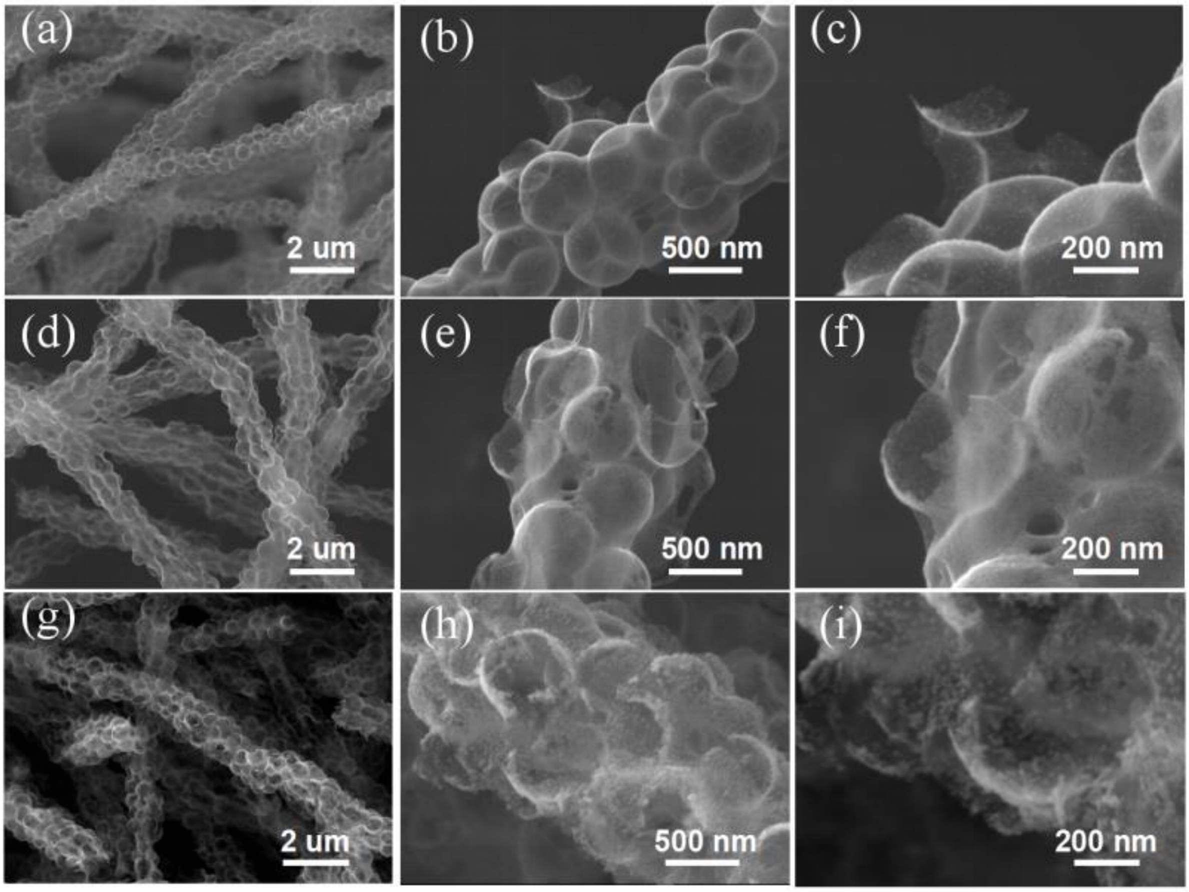

Figure S7. SEM images of (a-c) Co@N-C/PCNF-50 mM; (d-f) Co@N-C/PCNF-100 mM; (g-i) Co@N-C/PCNF-300 mM.

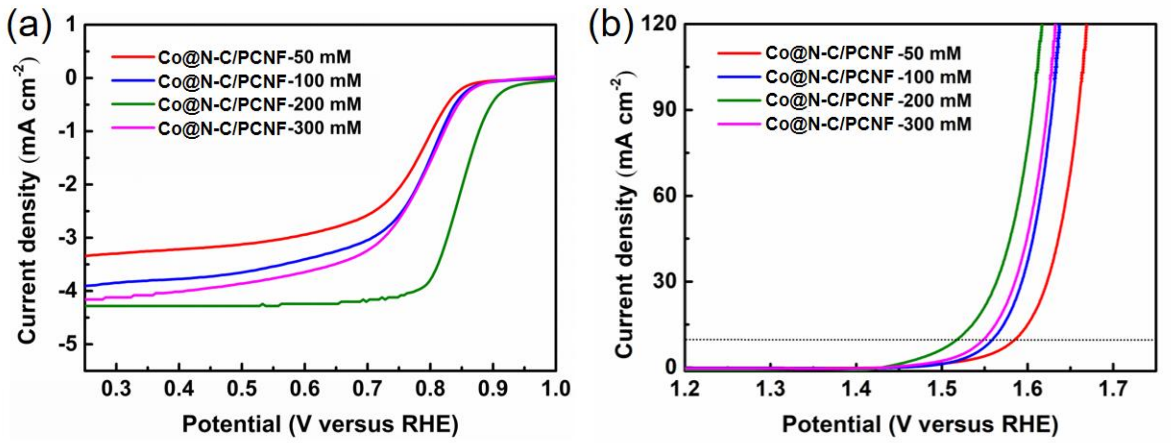

Figure S8. (a) ORR LSV curves and (b) OER LSV curves of Co@N-C/PCNF-M (M=50 mM, 100 mM, 200 mM, 300 mM).

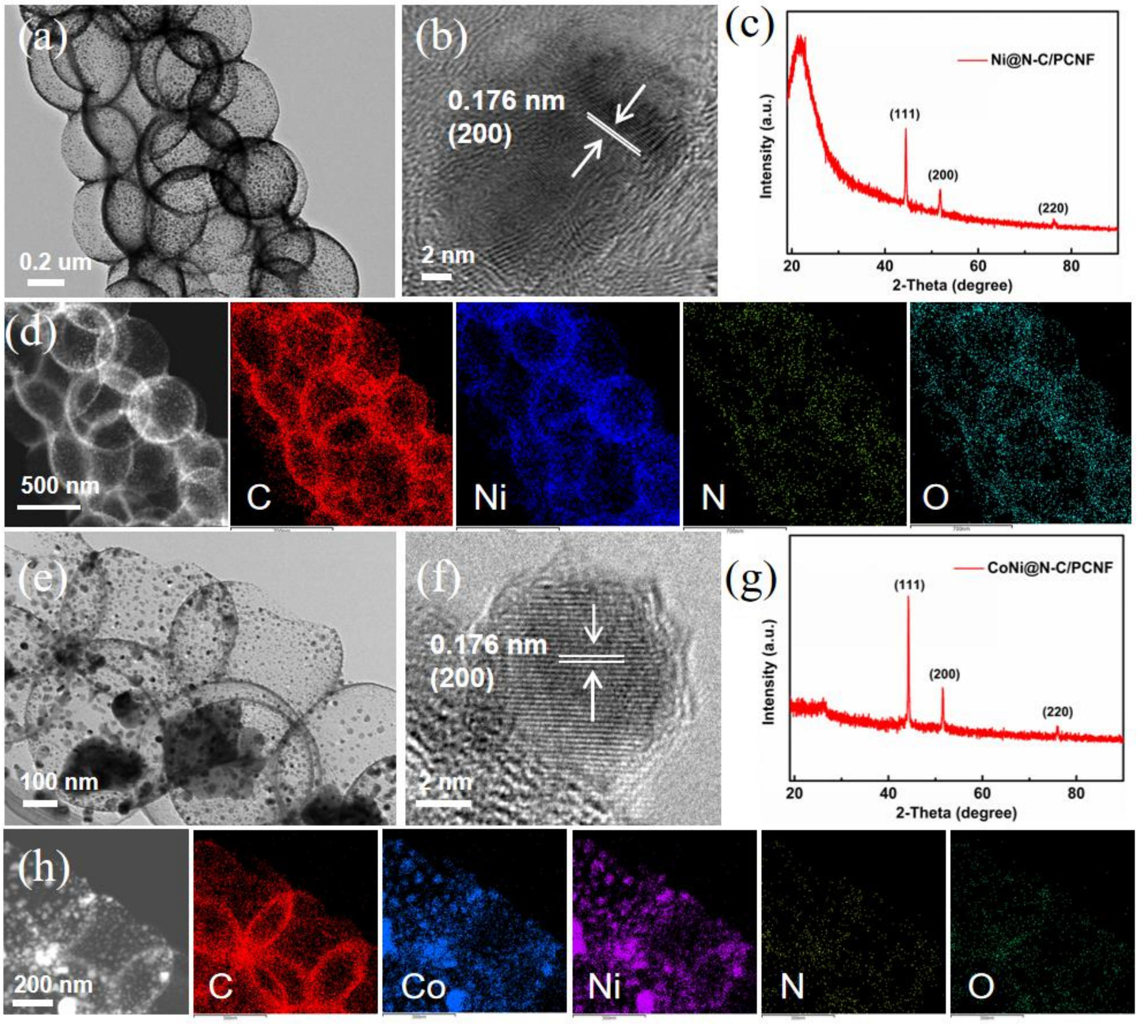

Figure S9. (a) TEM image, (b) HRTEM image, (c) XRD patternand of Ni@N-C/PCNF. (d) HAADF image and elemental mapping distribution of Ni@N-C/PCNF composite. (e) TEM image, (f) HRTEM image, (g) XRD patternand of Ni@N-C/PCNF, (h) HAADF image and elemental mapping distribution of CoNi@N-C/PCNF composite.

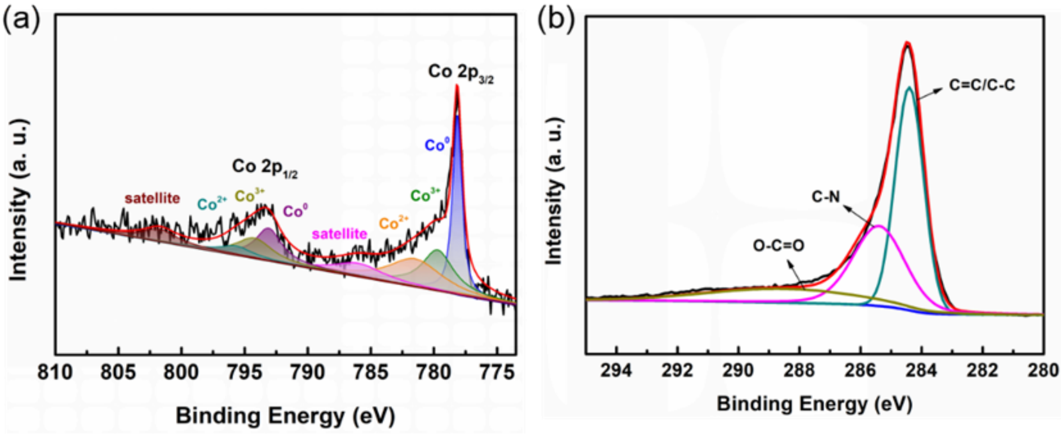

Figure S10. XPS spectra of (a) Co 2p and (b) C 1s peaks in Co@N-C/PCNF.

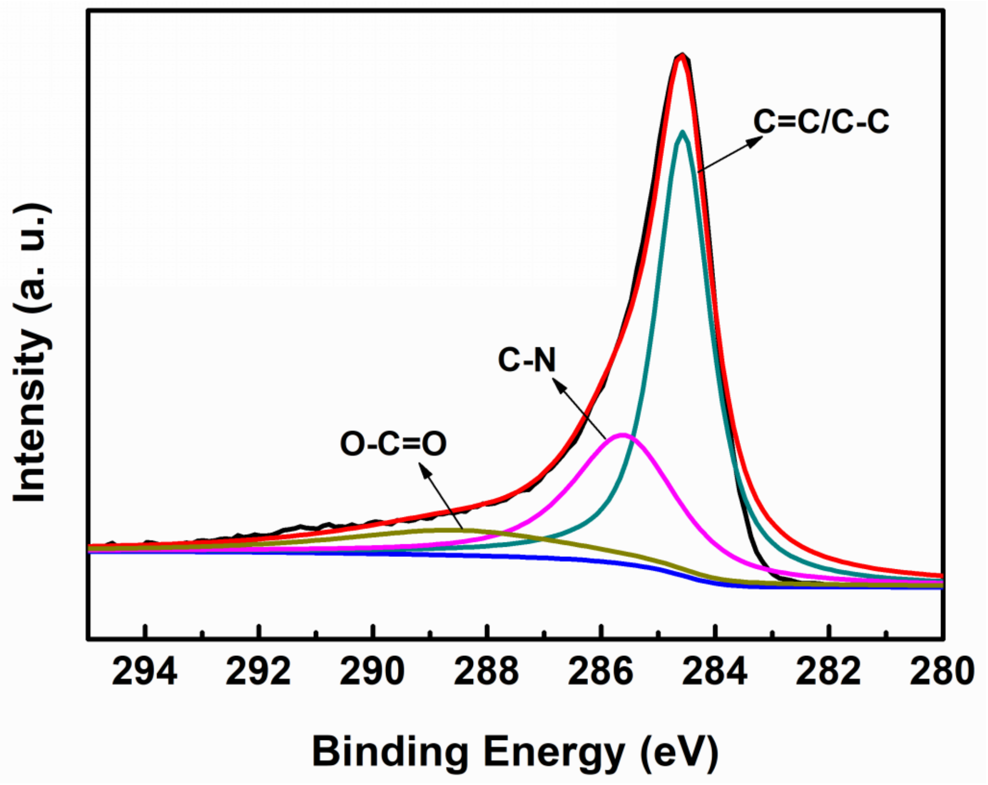

Figure S11. XPS spectra of the C 1s peak in PCNF.

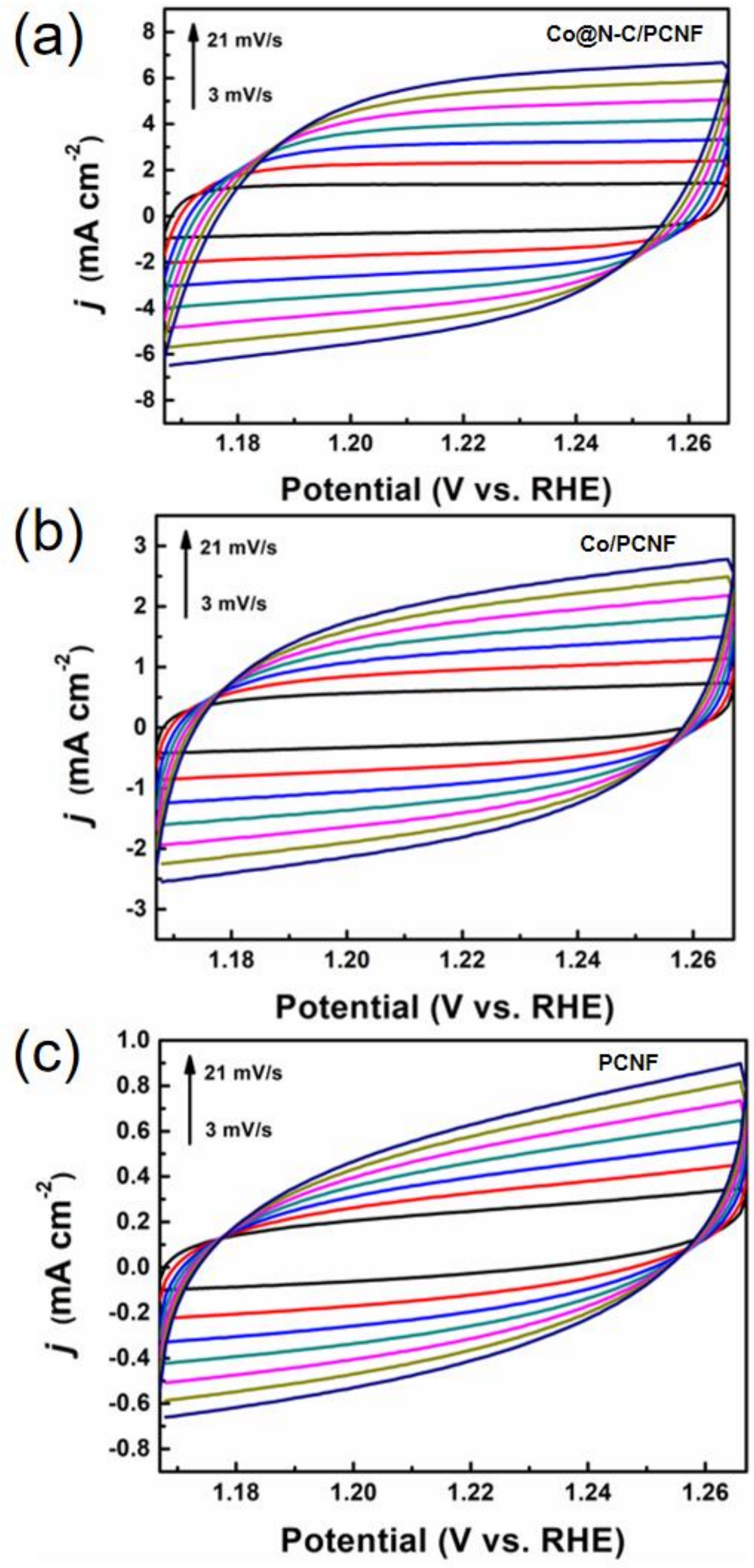

Figure S12. CV curves of (a) Co@N-C/PCNF, (b) Co/PCNF, and (c) PCNF at different scanning rates.

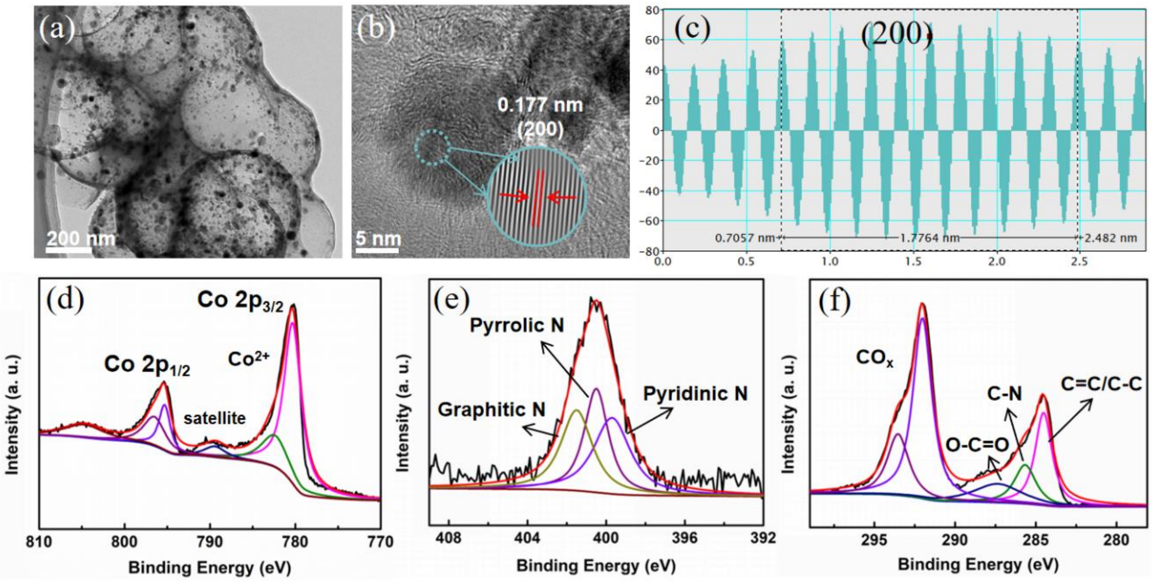

Figure S13. (a) TEM images, (b) HRTEM of Co@N-C/PCNF after OER, (c) signal intensity profile obtained from the dotted line area of (b), (d) Co 2p, (e) N 1s, (f) C 1s of Co@N-C/PCNF after OER.

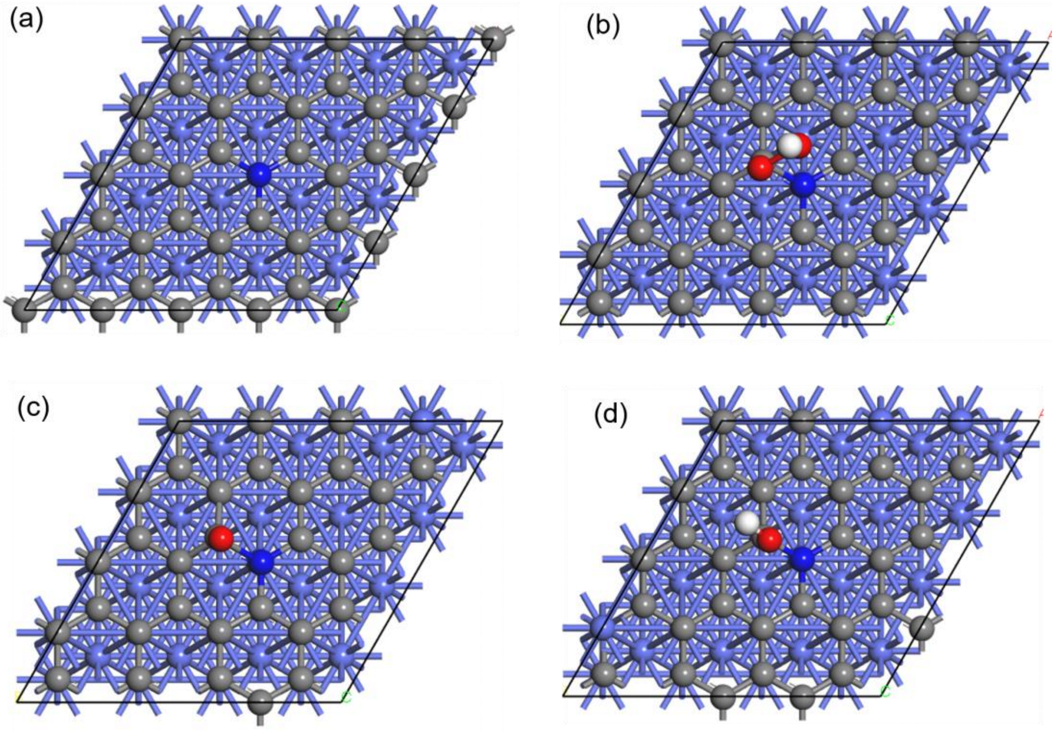

Figure S14. (a) The model of the lattice structure of Co@N-C. (b) The optimized atomic configurations of oxygen intermediates OOH*, (c) O*, and (d) OH* adsorbed on Co-N-C moiety.

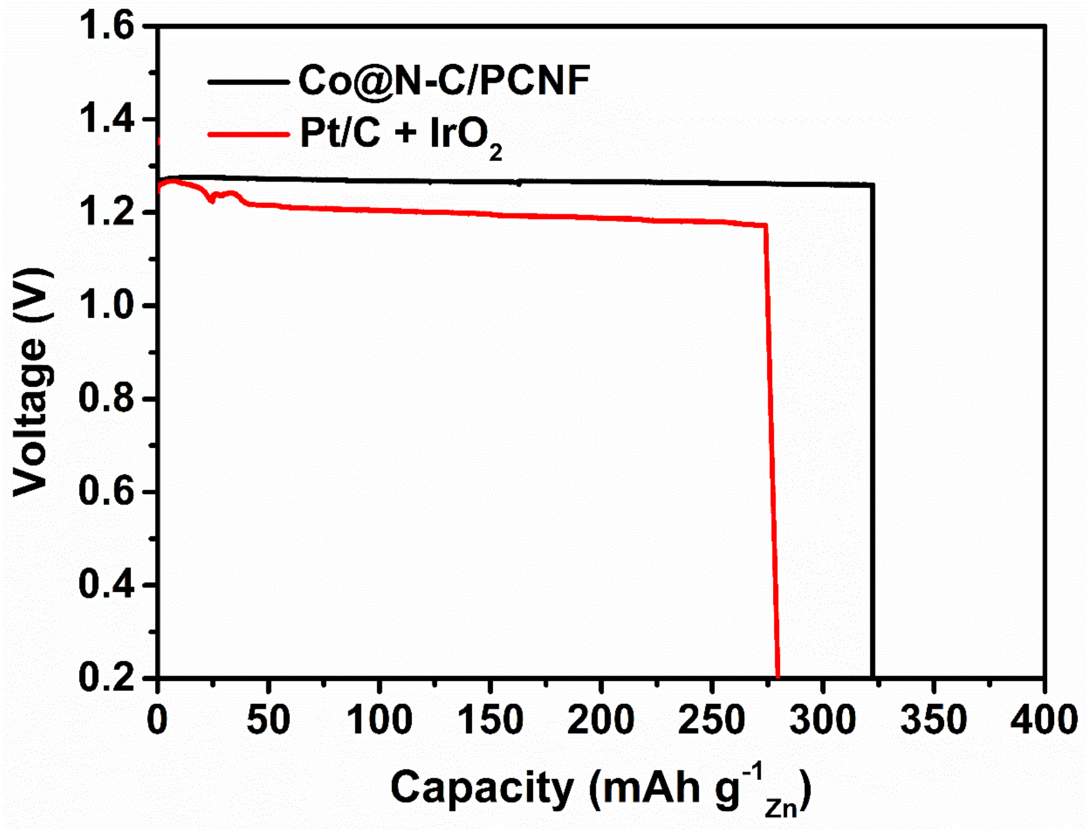

Figure S15. Voltage-capacity curves of AZAB using the Co@N-C/PCNF and Pt/C + IrO^2 as cathodes, respectively.

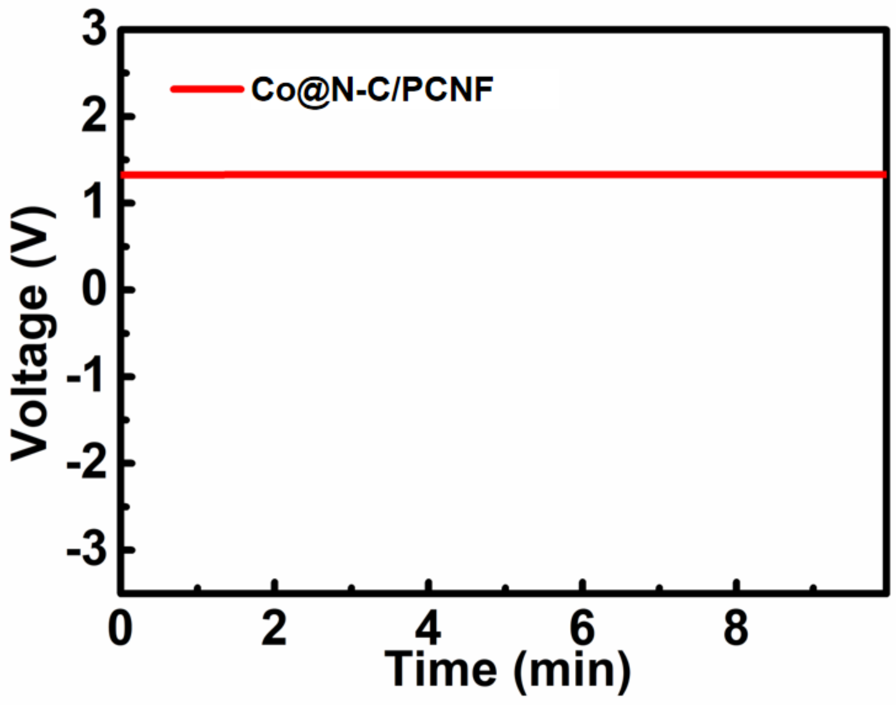

Figure S16. Open-circuit potential of the flexible ZAB using Co@N-C/PCNF as cathode.

Table S1. OER and ORR characteristics of electrocatalysts tested in this work.

	ORR		OER	
Catalyst	$E_{1/2}$ (V vs. RHE)	limiting diffusion current density (mA cm^{-2})	Overpotential 10 mA cm^{-2} (mV)	Overpotential 50 mA cm^{-2} (mV)
Co@N-C/PCNF	0.81 V	4.4	289	354
Co/PCNF	0.78 V	4.1	338	391
PCNF	0.71 V	3.3	384	465
IrO_2	-	-	377	455

Table S2. ORR properties of some recently reported electrocatalysts.

Catalyst	*Eo* (V vs. RHE)	$E_{1/2}$ (V vs. RHE)	References
Co_3O_4-CeO_2/Co-N-KB	0.93	0.80	13
Fe@N-CNT/HMCS	0.847	0.741	14
CoDNG900	0.942	0.827	15
H-Co@FeCo/N/C	1.03	0.91	16
Co/N-C@CNFs	0.95	0.85	17
Co_3O_{4-x}/NG	0.96	0.84	18
Co@NSCNT-900	0.91	0.83	19
Co-TA-800	0.95	-	20
CoSA/N, S-HCS	0.96	0.85	21
Co@N-C/PCNF	0.88	0.81	This work

Table S3. OER properties of some recently reported electrocatalysts.

Catalysts	Overpotential@ 10 mA cm^{-2} (mV)	Tafel slop ($mV\ dec^{-1}$)	References
Fe@N-CNT/HMCS	340	76	14
CoDNG900	405 (0.1 M KOH)	72	15
H-Co@FeCo/N/C	378 (0.1 M KOH)	95	16
Co_3O_{4-x}/NG	296	85	18
Co-TA-800	460 (0.1 M KOH)	66	20
CoSA/N, S-HCS	306	38	21
$Ni_{1.5}Co_{0.5}$@N-C NT/NF	243	103	22
Co-NCNT	370	56	23
Co/N-CNTs@ Ti_3C_2Tx	410	79	24
Co@N-C/PCNF	289	92	This work

Table S4. The electrochemical performance of flexible ZABs recently reported.

Catalysts	Open-circuit Voltage (OCV)	Cycling current density (mA cm^{-2})	Flexible ZABs Cycles / time	References
NiFe@N-CFs	1.18 V	1	about 60 cycles / more than 20 h	25
CoFe/N-HCSs	1.40 V	1	/ more than 10 h	26
$(Fe,Co,Ni)_9S_8$/NSCFs	1.435 V	5	about 140 cycles /	27
Ni/CNF-750	1.38 V	/	up to around 50 cycles / more than 5 h	28
FeP/Fe_2O_3@NPCA	1.42 V	5	50 cycles / 8 h	29
Co@N-C/PCNF	1.32 V	1	about 150 cycles / more than 24 h	This work

References

[1] Y. G. Wang, G. H. Zhao, X. J. Li, L. Liu, W. Cao and Q. Wei, *Biosens Bioelectron*. **2018**, *101*, 290.

[2] C. Liu, W. Zhou, J. Zhang, Z. Chen, S. Liu, Y. Zhang, J. Yang, L. Xu, W. Hu, Y. Chen and Y. Deng, *Adv. Energy. Mater*. **2020**, *10*, 2001397.

[3] C. Mikutta and R. Kretzschmar, *Environ. Sci. Technol*. **2011**, *45*, 9550.

[4] Z. Xia, H. Zhang, K. Shen, Y. Qu and Z. Jiang, *Physica B: Condensed Matter*. **2018**, *542*, 12.

[5] C. L. Li, M. C. Wu and R. Liu, *Appl. Catal. B-Environ*. **2019**, *244*, 150.

[6] M. D. Segall, P. J. D. Lindan, M. J. Probert, C. J. Pickard, P. J. Hasnip, S. J. Clark, M. C. Payne, J. Phys.: Condens. Matter. **2002**, *14*, 2717

[7] J. P. Perdew, K. Burke, M. Ernzerhof, *Phys. Rev. Lett.* **1996**, *77*, 3865.

[8] D. R. Hamann, M. Schlüter, C. Chiang, *Phys. Rev. Lett.* **197**9, *43*, 1494.

[9] S. Grimme, *J. Comput. Chem*. **2006**, *27*, 1787.

[10] S. K. Singh, K. Takeyasu, J. Nakamura, *Adv. Mater*. **2019**, 31, 1804297.

[11] Y. Wu, C. Li, W. Liu, H. Li, Y. Gong, L. Niu, X. Liu, C. Sun and S. Xu, *Nanoscale* **2019**, *11*, 5064.

[12] M. Bajdich, M. Garcia-Mota, A. Vojvodic, J. K. Norskov and A. T. Bell, *J Am Chem Soc*. **2013**, *135*, 13521.

[13] G. Z. Li, Y. C. Mu, Z. X. Huang, N. G. Wang, Y. Y. Chen, J. Liu, G. P. Liu, O. L. Li, M. H. Shao and Z. C. Shi, *Sci. China Mater*. **2021**, *64*, 73.

[14] J. Liu, H. Xu, H. Li, Y. Song, J. Wu, Y. Gong, L. Xu, S. Yuan, H. Li and P. M. Ajayan, *Appl. Catal. B: Environ*. **2019**, *243*, 151.

[15] A. Wang, C. Zhao, M. Yu and W. Wang, *Appl. Catal. B: Environ*. **2021**, *281*, 119514.

[16] Y.-j. Wu, X.-h. Wu, T.-x. Tu, P.-f. Zhang, J.-t. Li, Y. Zhou, L. Huang and S.-g. Sun, *Appl. Catal. B: Environ*. **2020**, *278*, 119259.

[17] H. J. Meng, Y. M. Liu, H. R. Liu, S. P. Pei, X. X. Yuan, H. Li and Y. M. Zhang, *ACS Appl. Mater. Interface* **2020**, *12*, 41580-41589.

[18] J. Qin, Z. Liu, D. Wu and J. Yang, *Appl. Catal. B: Environ.* **2020**, *278*, 119300.

[19] T. Oh, K. Kim and J. Kim, *J. Energy Chem.* **2019**, *38*, 60-67.

[20] J. Wei, Y. Liang, Y. X. Hu, B. Kong, J. Zhang, Q. F. Gu, Y. P. Tong, X. B. Wang, S. P. Jiang and H. T. Wang, *Angew. Chem. Int. Ed.* **2016**, *55*, 12470.

[21] Z. Zhang, X. Zhao, S. Xi, L. Zhang, Z. Chen, Z. Zeng, M. Huang, H. Yang, B. Liu, S. J. Pennycook and P. Chen, *Adv. Energy Mater.* **2020**, 10, 2002896.

[22] T. Li, S. Li, Q. Liu, J. Yin, D. Sun, M. Zhang, L. Xu, Y. Tang and Y. Zhang, *Adv. Sci.* **2020**, *7*, 1902371.

[23] J. Cong, C. Li, T. Zhao, J. Wu, R. Zhang, W. Ren, S. Wang, J. Gao, Y. Liu and J. Yao, *J. Solid State Chem.* **2017**, *253*, 227.

[24] Y. Zhang, H. Jiang, Y. Lin, H. Liu, Q. He, C. Wu, T. Duan and L. Song, *Adv. Mater. Interfaces.* **2018**, *5*, 1800392.

[25] Y. L. Niu, X. Teng, S. Q. Gong and Z. F. Chen, *J. Mater. Chem. A.* **2020**, *8*, 13725.

[26] J. M. Li, Y. M. Kang, W. L. Wei, X. Li, Z. Q. Lei, P. Liu, *Chem. Eng. J.* **2021**, *407*, 127961.

[27] T. Z. Jiang, P. Dai, W. Zhang, M. Z. Wu, *Electrochim. Acta.* **2021**, *373*, 137903.

[28] G. Q. Liu, X. Xia, C. J. Zhao, X. Zhang, W. X. Zhang, *J. Colloid Interface Sci.* **2021**, *588*, 627.

[29] K. Z. Wu, L. Zhang, Y. F. Yuan, L. X. Zhong, Z. X. Chen, X. Chi, H. Lu, Z. H. Chen, R. Zou, T. Z. Li, C. Y. Jiang, Y. K. Chen, X. W. Peng, and J. Lu, *Adv. Mater.* **2020**, *32*, 2002292.
